# MDCT of unique drainage of the anterior interventricular coronary vein into the left atrium: two case reports

**DOI:** 10.1259/bjrcr.20190062

**Published:** 2020-02-12

**Authors:** Amal Abdelsattar Sakrana, Shadha A. Ahmed Alzubaidi, Abdulhameed Mohmmed Shahat

**Affiliations:** 1 Madina Cardiac Center, 23411 AL Madinah Al munawwrah, Khaled Bin Al Waleed Road, Medina, Saudi Arabia; 2 Department of Diagnostic and Interventional radiology, Mansoura University Hospital, 35112 12 El-Gomhoreya street, Mansoura, Egypt

## Abstract

Pre-procedural CT mapping of the coronary venous system is advised by the current guidelines before many interventional procedures. The published literature for variants of the coronary venous system is scarce. The variant of anterior interventricular vein (AIV) drainage into the left atrium is extremely rare. In this paper, we present multidetector CT findings of two cases of anomalous drainage of the anterior interventricular vein into the left atrium.

## Introduction

CT mapping of the cardiac venous system (CVS) had attained an increasing value after the significant advance in the therapeutic interventional cardiac procedures such as; cardiac pacing, ablation of arrhythmias, retrograde cardioplegia perfusion (RCP), stem cell therapy, and targeted drug therapy.^1^ Successful targeted lead placement or ablation advised by the current guidelines is usually hindered by the individual anatomical variations of the cardiac veins.^2^ Many CVS congenital anomalies have been described, but the literature for variants of the coronary sinus (CS) tributaries is scarce.^3^ In this paper, we present multidetector CT (MDCT) findings of two cases of anomalous drainage of the anterior interventricular coronary vein into the left atrium.

## Case 1

A 46-years-old female patient complaining of atypical chest pain was referred to our radiology department to rule-out coronary artery disease. She has no hypertension, diabetes mellitus, and no history of smoking. Physical examination and echocardiography were normal. Cholesterol level was slightly elevated. Levels of cardiac enzymes were normal. Prospectively ECG-gated multidetector CT (MDCT) was performed on a 64-detector scanner (GE, Light Speed, VCT). Scanning parameters were; 120 kV, 350 mAs, 330 ms rotation time, 64 × 0.625 mm collimation, and 0.4 increments. The heart rate was 60 beats per minute during acquisition. 90 ml of iodinated contrast medium (CM) (Omnipaque-350; Daiichi Sankyo, Tokyo, Japan) was injected at a rate of 5 ml s^−1^ followed by a 20 ml saline bolus injected at the same injection rate as for the CM via a 20-gauge catheter inserted into an antecubital vein. The test-bolus technique was used.

**Figure 1.**
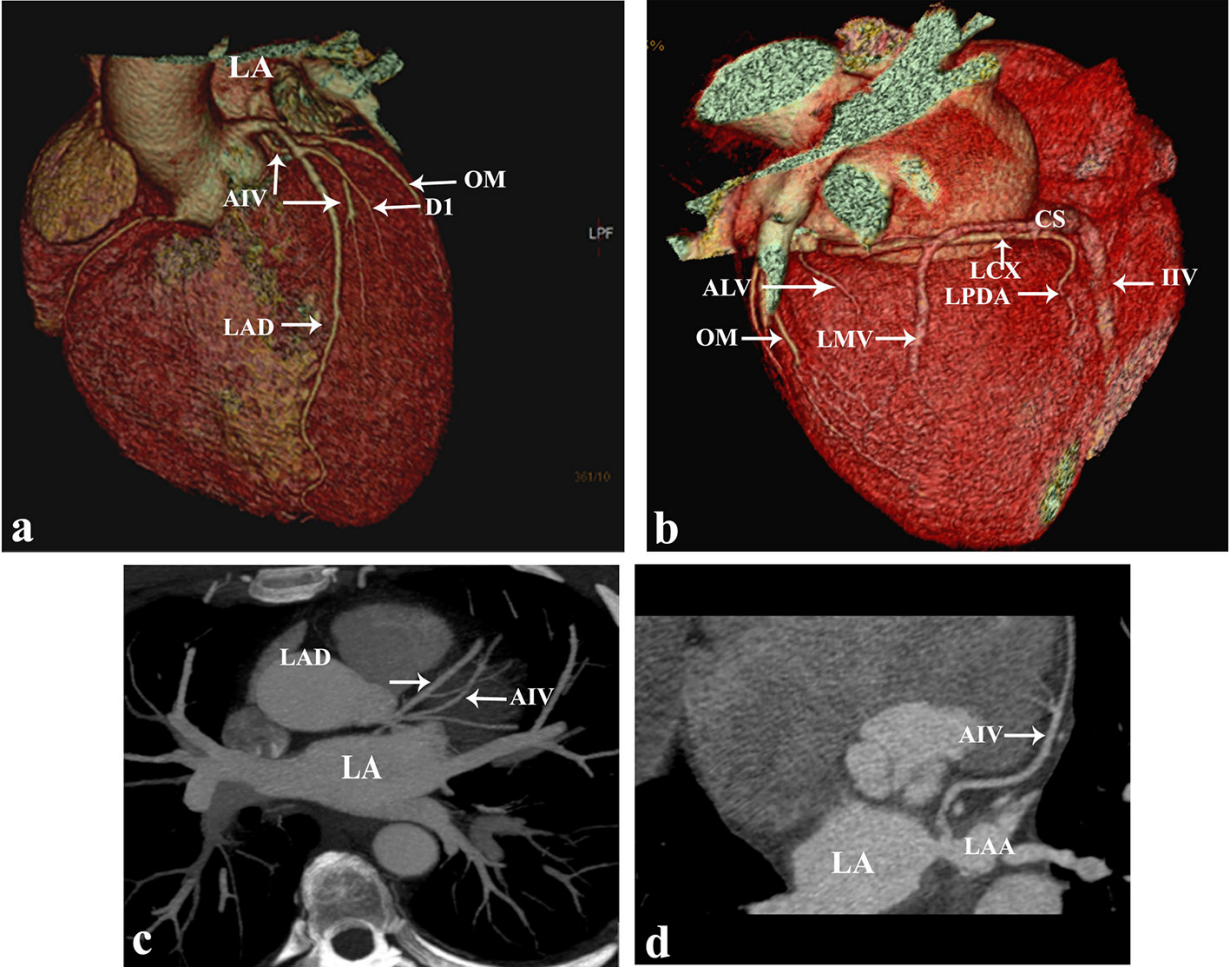
Multidetector coronary CT: three-dimensional volume rendering images (a, b). Oblique maximum intensity projection (MIP) image (c). Curved reformatted image (d). AIV passes parallel to LAD in the anterior interventricular sulcus deep to diagonal and left main arteries into the left atrium. Between AIV & IIV, there is ALV & LMV. AIV:anterior interventricular vein; CS: coronarysinus; D1: first diagonal artery; IIV: inferiorinterventricular vein; LAA: left atrial appendage; LA: left atrium; LAD: left anterior descending artery; Lcx: left circumflex artery; LPDA: left posteriordescending artery; OM: obtuse marginal artery; MIP: maximumintensity projection.

MDCT images (Figure 1) showed that anterior interventricular vein (AIV) originates in the middle third of the interventricular sulcus passing along with left anterior descending artery (LAD) deep to diagonal branch, and left main artery (LM|) to be drained into the left atrium. The proximal AIV diameter was 3.3 mm. AIV is seen draining the anterolateral vein (1.7 mm proximal diameter) that is connected to it at the proximal anterior part of the atrioventricular groove. The anterolateral vein sends a tiny tributary that passes in the atrioventricular sulcus [at the anatomical location of the great cardiac vein (GCV)] to be connected to a left marginal vein. Left marginal vein drains most of the left ventricular posterolateral wall with 3.7 mm proximal diameter. There is no posterolateral vein(s). CS is seen drained normally into the right atrium with a normal semicircular thebesian valve at the same level of the Eustachian valve just to the left of inferior vena cava (IVC) opening. The proximal CS diameters were (10 × 7.9 mm).

## Case 2

**Figure 2.**
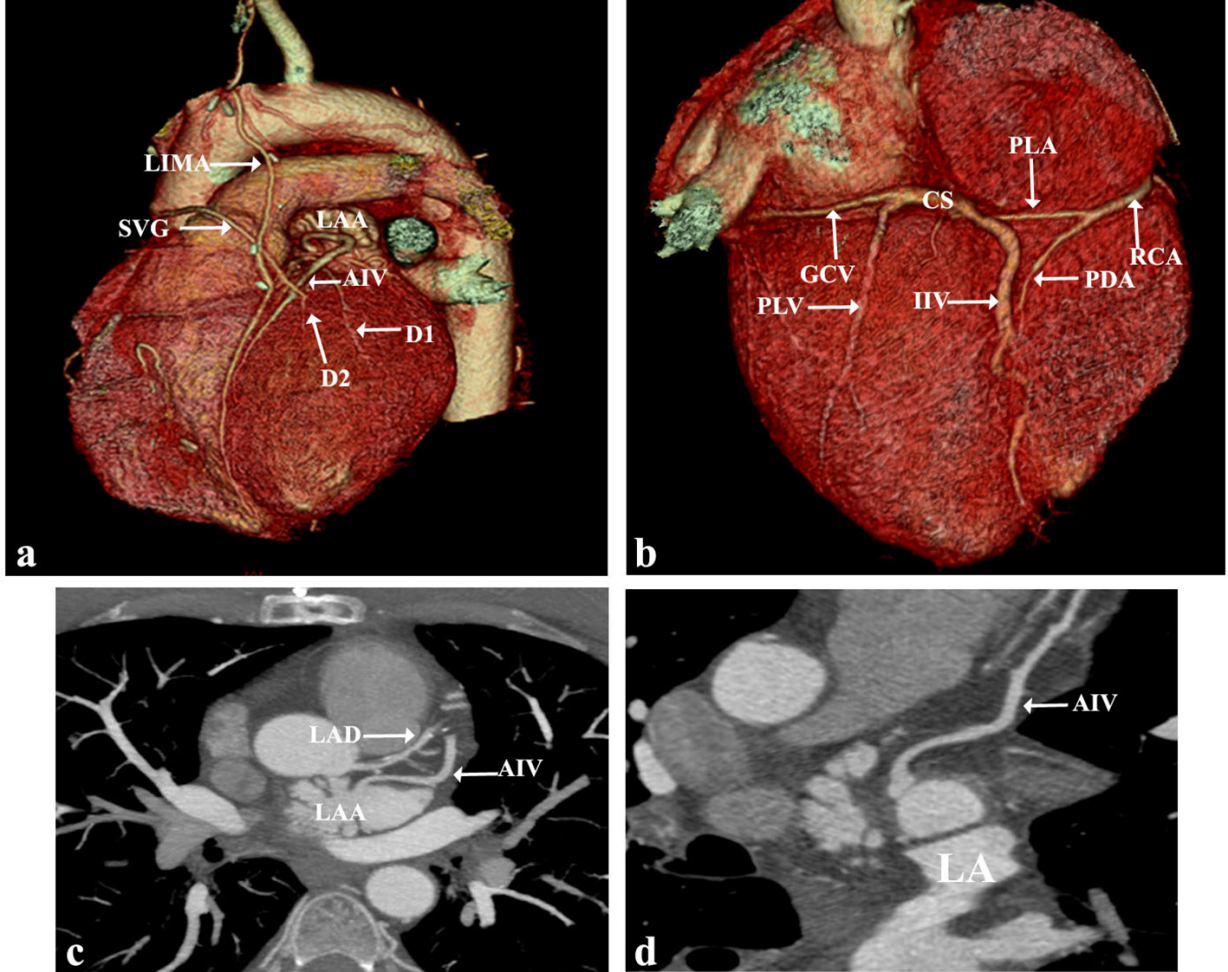
Multidetector coronary CT of a status post CABG: three-dimensional volume rendering images (a, b). Oblique MIP image (c). Curved reformatted image (d). AIV passes parallel to LAD in the anterior interventricular groove and superficial to the first diagonal artery into the left atrium. AIV is connected to GCV by a small venous tributary, and then the GCV is drained normally to CS. GCV: great cardiac vein; CABG: coronary artery bypass graft; AIV: anterior interventricular vein; CABG: coronaryartery bypass graft; GCV: great cardiac vein; IIV: inferior interventricular vein; LAA: left atrial appendage; LAD: left anterior descending artery; MIP: maximum intensity projection.

A 47-years-old male patient complained of shortness of breath and chest pain on exertion for two months. He had CABG surgery 2 years back. The patient was referred to our radiology department to evaluate the graft patency. Echocardiography revealed basal to mid inferior wall hypokinesia and mildly impaired left ventricular systolic function with 50% ejection fraction. Prospectively ECG-gated MDCT was performed on the same scanner using the same scanning parameters of the previously represented case apart from an increased field of view from the thoracic inlet to the base of the heart. MDCT images (Figure 2) showed anomalous drainage of AIV into the left atrium. AIV originates beyond the left ventricular apex having 3.9 mm proximal diameter and passes superficial to the first diagonal artery. AIV is connected to GCV by a small venous tributary, and then the GCV is drained normally to CS. GCV has 3.9 mm proximal diameter.

Between AIV and inferior interventricular vein (IIV), only one posterolateral vein is seen of 4.9 mm proximal diameter drained into CS (23 mm) proximal to the thebesian valve. IIV proximal diameter is 4.8 mm. No anterolateral vein or left marginal vein. CS is drained into the right atrium above the Eustachian valve level and to the left of IVC opening. CS diameters were (12 × 5.9 mm).

## Discussion

The cardiac veins had been anatomically classified into two main groups: the greater cardiac venous system draining the external two-thirds of the ventricular myocardium, and the lesser CVS consisting of the thebesian vessels draining the internal third.^3^ The greater CVS includes coronary sinus and non-coronary sinus tributaries. The main CS tributaries are: AIV, GCV, the left marginal vein, the posterior vein (posterolateral vein of the left ventricle), and the middle cardiac vein (IIV).^1,4^ The AIV ascends in the anterior interventricular sulcus parallel to LAD, and then enters the left atrioventricular groove where it is defined as the GCV. The GCV ends ultimately in the CS. The transition point from the GCV and CS is demarcated by the oblique vein of Marshall.^3^ The left marginal vein courses along the lateral aspect of the left ventricle along the obtuse marginal artery and drains into the GCV or directly into the CS. The posterolateral veins of the left ventricle are one to three in number and drain in most cases (75%) into the CS, but they can be drained into GCV.^3,5^ IIV or the middle cardiac vein runs in the posterior interventricular sulcus parallel to the posterior descending coronary artery and drains into the CS just proximal to its termination in the right atrium.^6^

Recently, many cardiac interventions need pre-procedural detailed assessment of the coronary venous system that is now feasible by MDCT as a non-invasive imaging modality. MDCT is as accurate as retrograde venography to depict CS and its tributaries.^7^

Transvenous pacemaker implantation is contraindicated for patients with previous mechanical tricuspid valve replacement with a potential risk of tricuspid valve malfunction and tricuspid regurgitation. In those cases, left ventricular pacing can be performed through AIV.^8^ Yoda et al presented a case of successful left ventricular pacing through AIV after preceding mechanical tricuspid, mitral and aortic valve replacements.^9^

Herein, we represent two cases of rare AIV drainage into the left atrium, and the rest of the cardiac veins drain into the right atrium normally. According to our knowledge, there were three publications of a similar drainage manner.^10–12^ Another reported case of anomalous drainage of all coronary veins and a remnant coronary sinus into the left atrium with persistent left superior vena cava was described.^13^

## Learning points

- Radiologists and interventional cardiologists should be aware of the unique anomalous drainage of the anterior interventricular vein into the left atrium especially, for patients with previous mechanical tricuspid valve replacement.
- MDCT is a proper non-invasive imaging modality for diagnosis of the coronary venous system anomalies as accurate as retrograde venography.
